# Tissue-specific populations from amniotic fluid-derived mesenchymal stem cells manifest variant in vitro and in vivo properties

**DOI:** 10.1007/s13577-023-01008-z

**Published:** 2023-12-12

**Authors:** Nengqing Liu, Yi Cheng, Ding Wang, Hongmei Guan, Diyu Chen, Juan Zeng, Dian Lu, Yuanshuai Li, Yinghong Yang, Qian Luo, Lifen Zhu, Bin Jiang, Xiaofang Sun, Bing Song

**Affiliations:** 1 Department of Obstetrics and Gynecology, Guangdong Provincial Key Laboratory of Major Obstetric Diseases, Guangdong Provincial Clinical Research Center for Obstetrics and Gynecology, Guangdong-Hong Kong-Macco Greater Bay Area Higher Education Joint Laboratory of Maternal-Fetal Medicine, The Third Affiliated Hospital of Guangzhou Medical University, Guangzhou, 510005 China; 2 Guangzhou Regenerative Medicine and Health Guangdong Laboratory, Guangzhou, 510005 China; 3 Department of Orthopedics, Shenzhen Intelligent Orthopaedics and Biomedical Innovation Platform, Shenzhen Second People’s Hospital, Shenzhen, 518035 China

**Keywords:** Amniotic fluid, Heterogeneity, Mesenchymal stem cells, Sepsis

## Abstract

Amniotic fluid derived mesenchymal stem cells (AFMSCs), shed along the fetal development, exhibit superior multipotency and immunomodulatory properties compared to MSCs derived from other somatic tissues (e.g., bone marrow and fat). However, AFMSCs display heterogeneity due to source ambiguity, making them an underutilized stem cells source for translational clinical trials. Consequently, there is an urgent need to identify a method to purify the AFMSCs for clinical use. We found that the AFMSCs can be categorized into three distinct groups: kidney-specific AFMSCs (AFMSCs-K), lung-specific AFMSCs (AFMSCs-L), and AFMSCs with an undefined tissue source (AFMSCs-X). This classification was based on tissue-specific gene expression pattern of single cell colony. Additionally, we observed that AFMSCs-X, a minority population within the AFMSCs, exhibited the highest multipotency, proliferation, resistance to senescence and immuno-modulation. Our results showed that AFMSCs-X significantly improved survival rates and reduced bacterial colony forming units (CFU) in cecal ligation and puncture (CLP)-induced septic mice. Therefore, our study introduces a novel classification method to enhance the consistency and efficacy of AFMSCs. These subpopulations, originating from different tissue source, may offer a valuable and innovative resource of cells for regenerative medicine purposes.

**Supplementary Information:**

The online version contains supplementary material available at 10.1007/s13577-023-01008-z.

## Background

Mesenchymal stem cells (MSCs) are somatic stem cells characterizing self-renewal and multipotency, and are widely used in cell therapy, tissue engineering, and regenerative medicine. AFMSCs are isolated from amniotic fluid (AF), exhibit characteristics as promising sources for stem therapy [1, 2]. Compared with the other somatic tissues (i.e., bone marrow and fat) derived MSCs, AFMSCs are easily obtained, free of ethical concerns, and importantly, have little pain for the donors. Moreover, the AFMSCs shed from the fetal development, show excellent multipotency but none tumorigenicity, extensive immunomodulatory properties [3, 4], and possess an intermediate state between embryonic and matured somatic cells [5]. Furthermore, AFMSCs have shown promising therapeutic potential in animal models of degenerative and inflammatory diseases affecting multiple tissues and organs [6]. However, AFMSCs have considerable heterogeneity which make it become an under-utilized stem cells source for clinical trials.

To address the concern of heterogeneity of AFMSCs, many groups have investigated methods for identification of the subpopulation of AFMSCs or enrich the subtype of AFMSCs with surface markers. Roubelakis MG et al. found that early colonies of AF-mesenchymal progenitor cells (AF-MPCs) consisted of two morphologically distinct adherent cell types, termed as spindle-shaped (SS) and round-shaped (RS) and found that SS-AF-MPCs express more CD90 and increased potential for proliferation and differentiation [7]. In parallel, comparative studies by Pipino found that Epithelial-like (E-like) and Fibroblast-like (F-like) AFMSCs phenotypes had different proteomic expression profiling [5]. However, those classification methods are mainly based on the morphology of AFMSCs which seem to be subjective. Latterly, molecular biological technique has been applied to identify the subtype of AFMSCs. Sacco et al. used expression of tissue-specific genes to identify renal, lung, cardiopulmonary, liver, bone marrow and mesenchymal AFMSCs in the 15–20 week AF [8]. Among these AFMSCs, nephrogenic AFMSCs were the predominant type, and expression of tissue-specific genes changed over time during pregnancy [9–11]. Besides, immunolabeling for CD117 (which is a transmembrane receptor tyrosine kinase for the stem cell factor encoded by the proto-oncogene c-kit) have been developed to screen and enrich AFMSCs which are supposed to be having high differentiation potential [12]. However, this approach raises concerns that CD117^+^ cells only make up less than 1% of the total AFMSCs population, and the enrichment process is complex and expensive for clinical application. Therefore, there is an urgent need to increase the homogeneity of AFMSCs through new potential avenues for translational research.

In this study, we cultured second-trimester AF samples (16–24 week) to address AFMSCs heterogeneity and identify tissue-sources subtypes. To enhance subtype accuracy, we isolated single-cell clones at passage 0, categorizing kidney-specific, lung-specific, and null-specific AFMSCs based on tissue-specific gene expression. These subclones were thoroughly evaluated for morphology, proliferation, cellular senescence, surface marker expression, differentiation, migratory ability, chemotaxis, and immunosuppressive capacity. Additionally, the newly identified AFMSCs subtype demonstrated anti-inflammatory effects on CLP-induced sepsis mice. We introduced a novel cost-effective and objective single-cell cloning-based classification method for AFMSCs. These findings enhance our understanding of AFMSCs’ biological characteristics and advance their potential for clinical applications.

## Results

### Isolation and identification of tissue-specific AFMSCs single clones

**Fig. 1:**
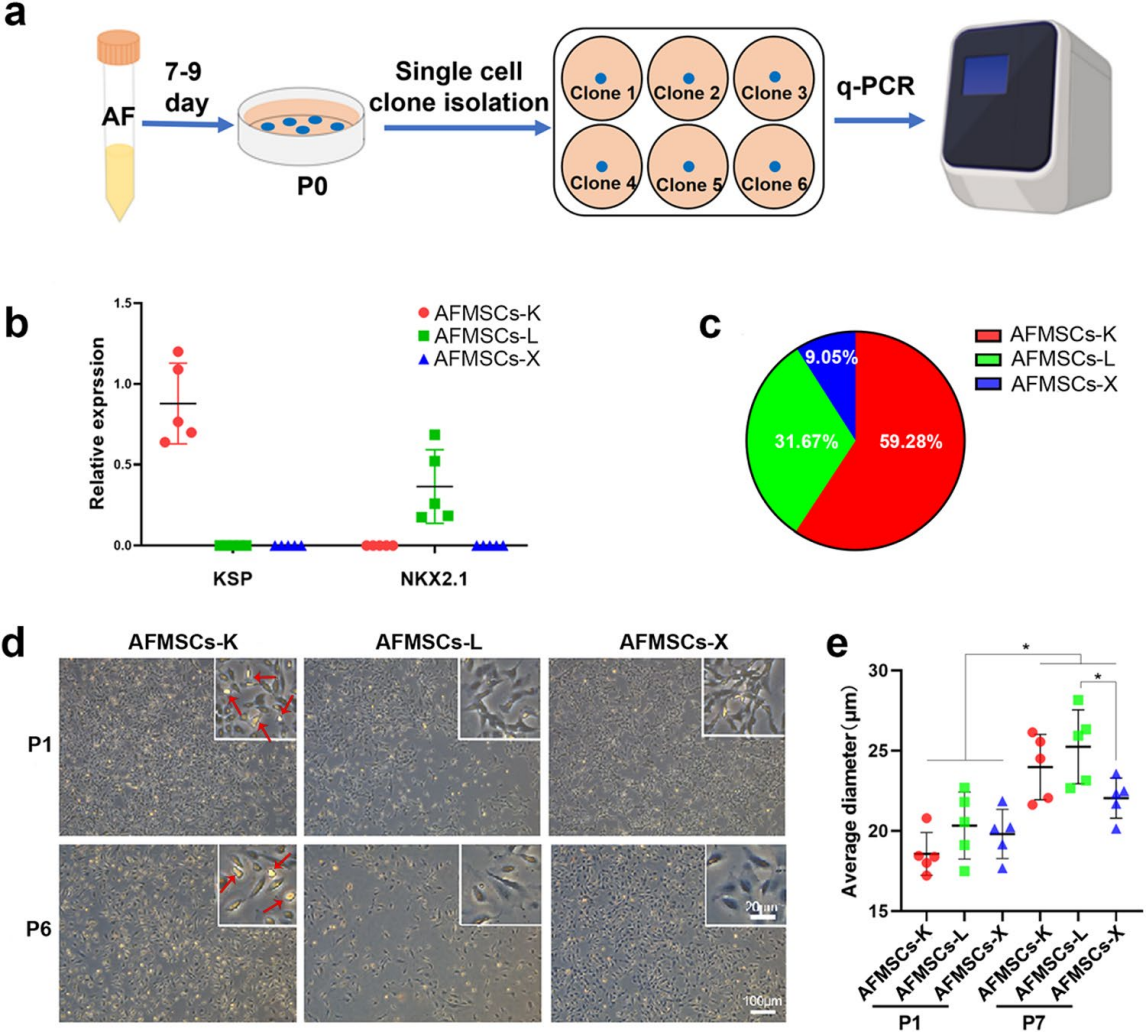
AFMSCs can expand as single-cell colonies and identified into three subtypes. **a** Sketch of single cell cloning of AF cells and identifying their tissue specificity (Day 7–9, P0) with tissue-specific gene expression pattern. **b** Expression of *KSP* and *NKX2.1* in AFMSCs clones formed by single cells, including *KSP*^+^/*NKX2.1*^−^ (kidney-specific AFMSCs, AFMSCs-K), *KSP*^−^/*NKX2.1*^+^ (lung-specific AFMSCs, AFMSCs-L), and *KSP*^−^/*NKX2.1*^−^ (unknown tissue-specific AFMSCs, AFMSCs-X). **c** The proportion of AFMSCs-K, AFMSCs-L, and AFMSCs-X clones according to gene expression of *KSP* and *NKX2.1* (*n* = 221). **d** Morphology of tissue-specific AFMSCs on the passage 1 and 6. Red arrows indicate cells with high refractivity. **e** The average diameter of the single AFMSCs on passage 1 and passage 7 (*n* = 5, *p < 0.05)

A total of 221 primary single cell clones were isolated from 10 donors after 7–9 days. Tissue-specific genes *KSP* (kidney) and *NKX2.1* (lung) were evaluated using RT-qPCR (Fig. 1 a). These clones can categorize into renal tissue-specific AFMSCs (*KSP*^+^/*NKX2.1*^−^, AFMSCs-K), lung tissue-specific AFMSCs (*KSP*^−^/*NKX2.1*^+^, AFMSCs-L), and null-specific AFMSCs (*KSP*^−^/*NKX2.1*^−^, AFMSCs-X) (Table 1, Fig. 1 b). Only 20 clones are *KSP*^*−*^*/NKX2.1*^*−*^ from three donors and are defined as AFMSCs-X clones, accounting for 9.05% of the total clones (Table 1, Fig. 1 c). AFMSCs-K is the largest population which was consistent with previous studies [9–11], followed by AFMSCs-L, and AFMSCs-X (Fig. 1 c). Moreover, the cells of each group show a unique morphology under inverted phase contrast microscope. Briefly, AFMSCs-K displayed an oval-like profile with high refractivity, AFMSCs-L exhibited a triangular shape with low refractive index, and AFMSCs-X showed a fibrillar-like profile. We have added red arrows to the high refractivity position of AFMSCs-K (Fig. 1 d). Meanwhile, the cells of each group show different sizes, and the average diameter gradually increased with continuous passage (Fig. 1 e).

**Table 1:** The patients’ information and composition of tissue-specific subclones derived from AFMSCs

Sample	Age	Gestational Age	Karyotype	AFMSCs-K subclones	AFMSCs-L subclones	AFMSCs-X subclones	Total subclones
1	32	16 + 2	46, XX	15	8	9	32
2	37	22 + 6	46, XY	10	6	0	16
3	37	23 + 2	46, XX	7	4	0	11
4	26	18 + 1	46, XY	20	10	0	30
5	35	16 + 6	46, XY	8	4	5	17
6	26	17 + 4	46, XX	15	7	0	22
7	26	22 + 3	46, XY	17	7	0	24
8	32	16 + 1	46, XY	10	7	6	23
9	31	23 + 5	46, XX	12	8	0	20
10	42	16 + 2	46, XY	17	9	0	26
Sum of subclones				131	70	20	221
Percentage of subclones				59.28%	31.67%	9.05%	100%

### AFMSCs-X show the best proliferation and delayed senesce

**Fig. 2:**
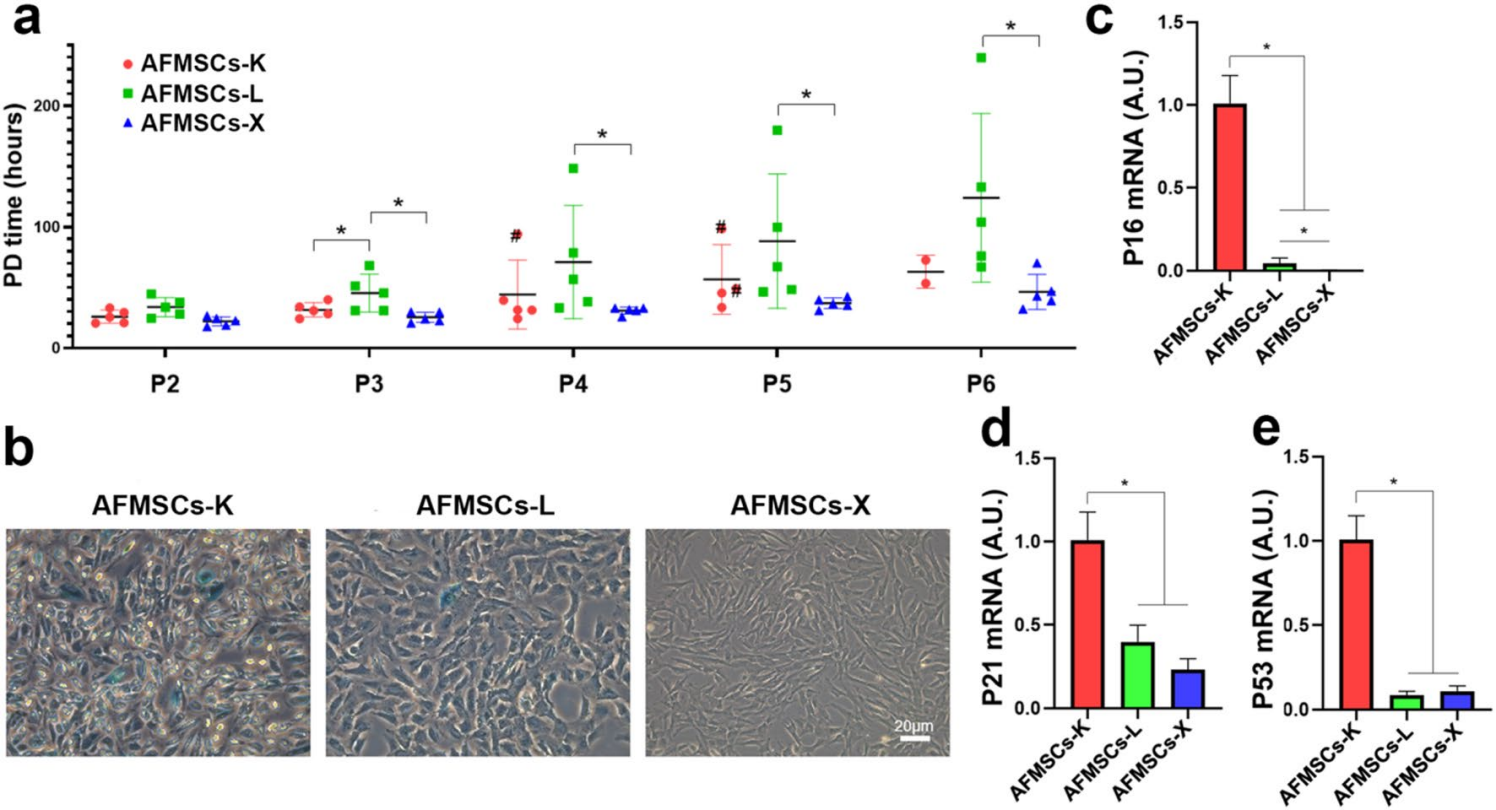
The AFMSCs-X shows better cell proliferation ability and delayed cell senescence than the other two subtypes. **a** Cell population doubling time (PDT) of different tissue-specific AFMSCs was assayed from passage 2 to passage 6 (*n* = 5, *p < 0.05, # means that the cell stopped proliferating); **b** Senescence-related (SA) β-galactosidase^+^ cell staining (indicated by red arrow) performed to detect the senescence in P3 AFMSCs; **c**–**e** Expression of genes involved in cell cycle-regulation (*P16*, *P21*, and *P53*) in different tissue-specific AFMSCs (*n* = 3, *p < 0.05)

Population doubling time (PDT) was used to evaluate the proliferative capacity of AFMSCs from P2 to P6. Our results indicated that AFMSCs-X showed a significantly shorter PDT (Fig. 2 a). AFMSCs-K cultures contained the highest number of SA-β gal-positive cells, AFMSCs-L contained fewer, while AFMSCs-X barely contained SA-β gal-positive cells (Fig. 2 b). Accordingly, RT-qPCR results showed that the senescence-specific genes (*P16*, *P21*, and *P53*) have the highest expression in AFMSCs-K, medium in the AFMSCs-L, and the least in the AFMSCs-X (Fig. 2 c-e). Meanwhile, immunofluorescence results showed that all three tissue-specific AFMSCs express embryonic stem cells (ESCs) markers: OCT4, SOX2, NANOG, and SSEA4 (Supplementary Fig. 1), which were consistent with the flow cytometry results (Supplementary Fig. 2). Presumably, AFMSCs-X have the best proliferation and delayed senescence than AFMSCs-K and AFMSCs-L which may partially coordinate its high expression of pluripotent marker SSEA-4 (Supplementary Fig. 2. a, e).

### Tissue-specific AFMSCs show specific MSCs marker expression profiles

**Fig. 3:**
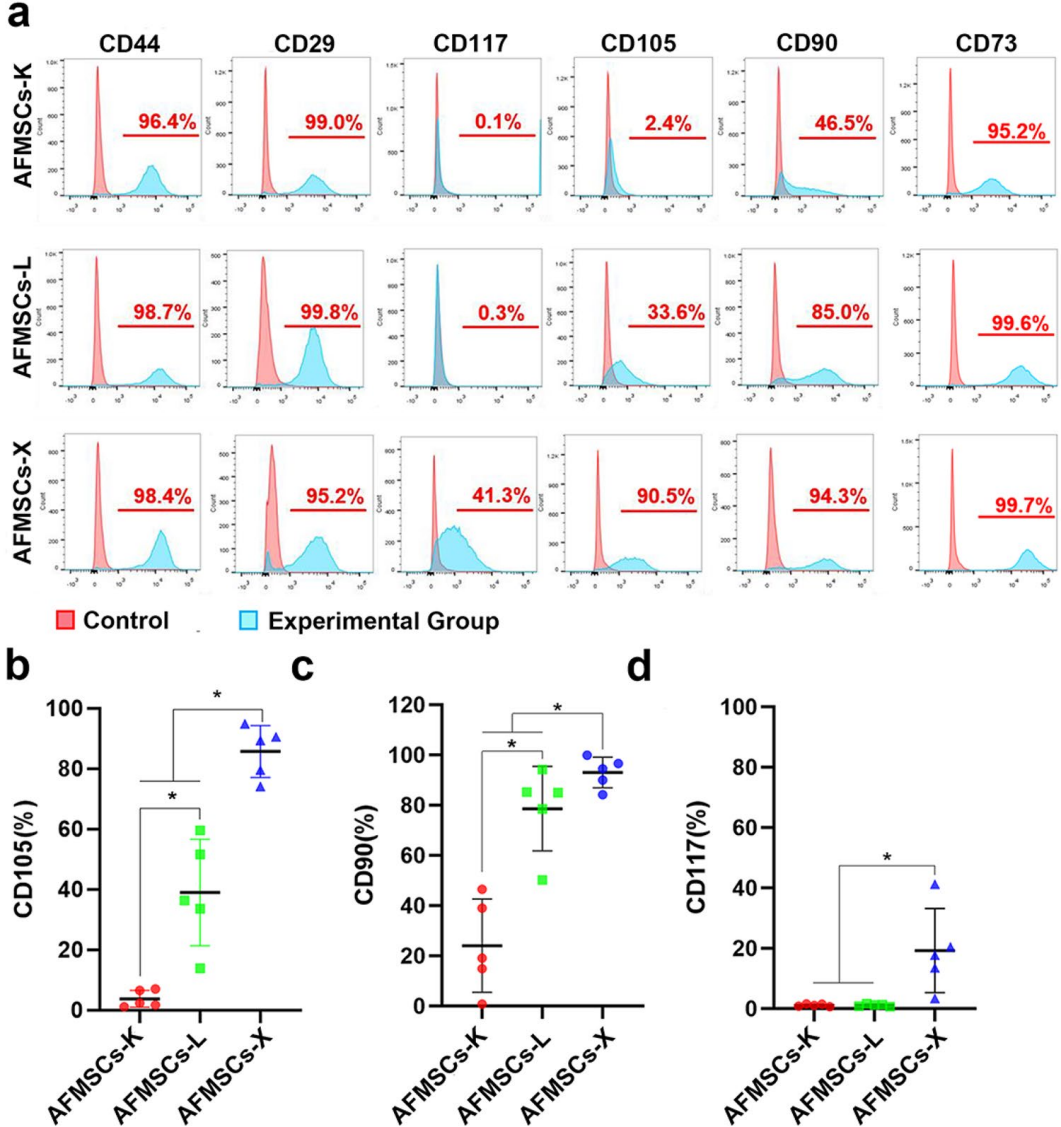
The AFMSCs-X expressed higher MSC-positive markers than the other two subtypes. **a** Representative flow cytometry plots of the three subtypes of AFMSCs, which are positive on the MSC-positive markers, including CD44, CD29, CD117, CD105, CD90, and CD73. And the statistics of expression of CD105, CD90, and CD117 of all the three types of AFMSCs are separately plotted as shown as (b)-(d) with mean ± SD (*n* = 5, *p < 0.05)

Minimal criteria for identifying multipotent MSCs used to evaluate three tissue-specific AFMSCs with flow cytometry[13]. The positive markers for MSCs showed that tissue-specific AFMSCs expressed high levels of CD44, CD29, and CD73 with no significant differences (Fig. 3 a). In contrast, there were significant differences in CD105, CD90, and CD117 expression among the three groups (*n* = 5) (Fig. 3 b-d). Both CD105 and CD90 expression were lowest in AFMSCs-K, moderate in AFMSCs-L, and highest in AFMSCs-X (Fig. 3 b, c). However, AFMSCs-K and AFMSCs-L were negative for CD117 expression, while AFMSCs-X expressed a low level of CD117 (19.31 ± 13.90%) (Fig. 3 d). About the negative markers (i.e., hematopoietic stem cells markers cocktail) for MSCs show that AFMSCs-K, AFMSCs-L, or AFMSCs-X little express CD19, HLA-DR, CD34, CD45, or CD11b (Supplementary Fig. 3). Therefore, all three tissue-specific AFMSCs match the minimal criteria for defining MSCs.

### AFMSCs-X showed the highest differentiation potential in vitro

**Fig. 4:**
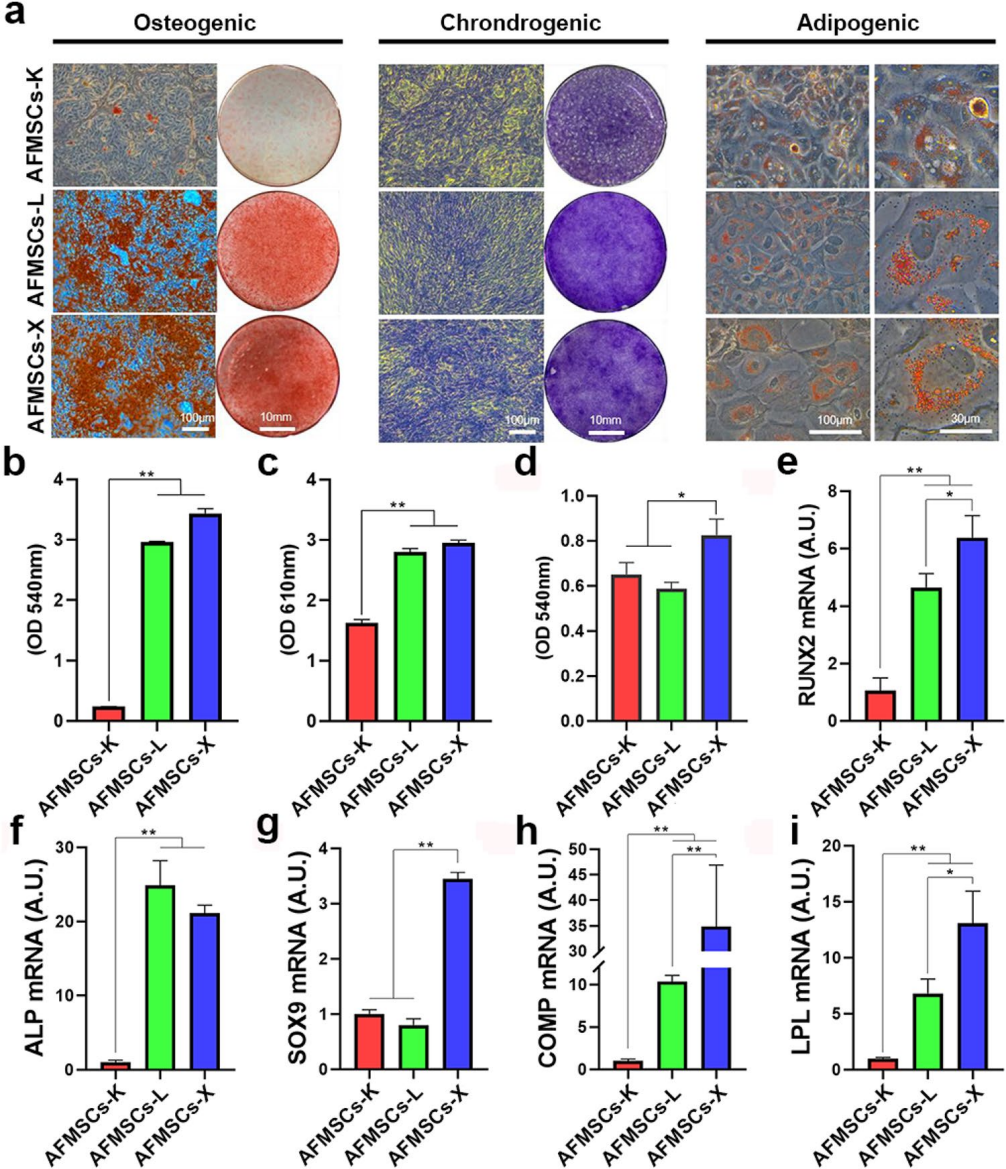
The AFMSCs-X shows better differentiation capabilities into trilineage than the other two subtypes. **a** Cell-specific staining for osteocytic, chondrocyte and adipocytic differentiation in different tissue-specific AFMSCs; **b**–**d** Quantitative analysis of tissue-specific staining in different tissue-specific AFMSCs (*n* = 3, *p < 0.05, **p < 0.01). (e)-(j) RT-qPCR was used to assay mRNA expression of tissue-specific markers as follows: *RUNX2* and *ALP* for osteocytic differentiation, *SOX9* and *COMP* for chondrocyte differentiation, and *LPL* for adipocytic differentiation (*n* = 3, *p < 0.05, **p < 0.01)

Trilineage differentiation potential (for osteogenesis, chondrogenesis, or adipogenesis) were evaluated to assess the multipotency of tissue-specific AFMSCs. They all showed typical calcium deposit, collagen deposit, and oil droplets. Which were positive with Alizarin red S, Toluidine blue, and Oil Red O staining, respectively (Fig. 4 a). Moreover, the absorbance results showed that the differentiation capacity of AFMSCs-X is significantly the best (Fig. 4 b-d). Furthermore, tissue-specific genes *RUNX2* and *ALP* (for osteocytes, Fig. 4 e, f), *SOX9* and *COMP* (for chondrocytes, Fig. 4 g, h), and *LPL* (for adipocytes, Fig. 4 i) were analyzed using RT-qPCR. The results indicated that AFMSCs-X showed the highest differentiation potential, followed by that AFMSCs-L and AFMSCs-K (Fig. 4 e-i). The tumor formation risk of tissue-specific AFMSCs was evaluated by soft agar colony formation assay (SAA) and teratoma formation assay. AFMSCs did not form tumor colonies or spheroids in vitro or teratomas in vivo, unlike 293 T tumor cells and ESCs controls, indicating no tumor formation risk (Supplementary Fig. 4).

### AFMSCs-X shows the best immunosuppressive effects in vitro

**Fig. 5:**
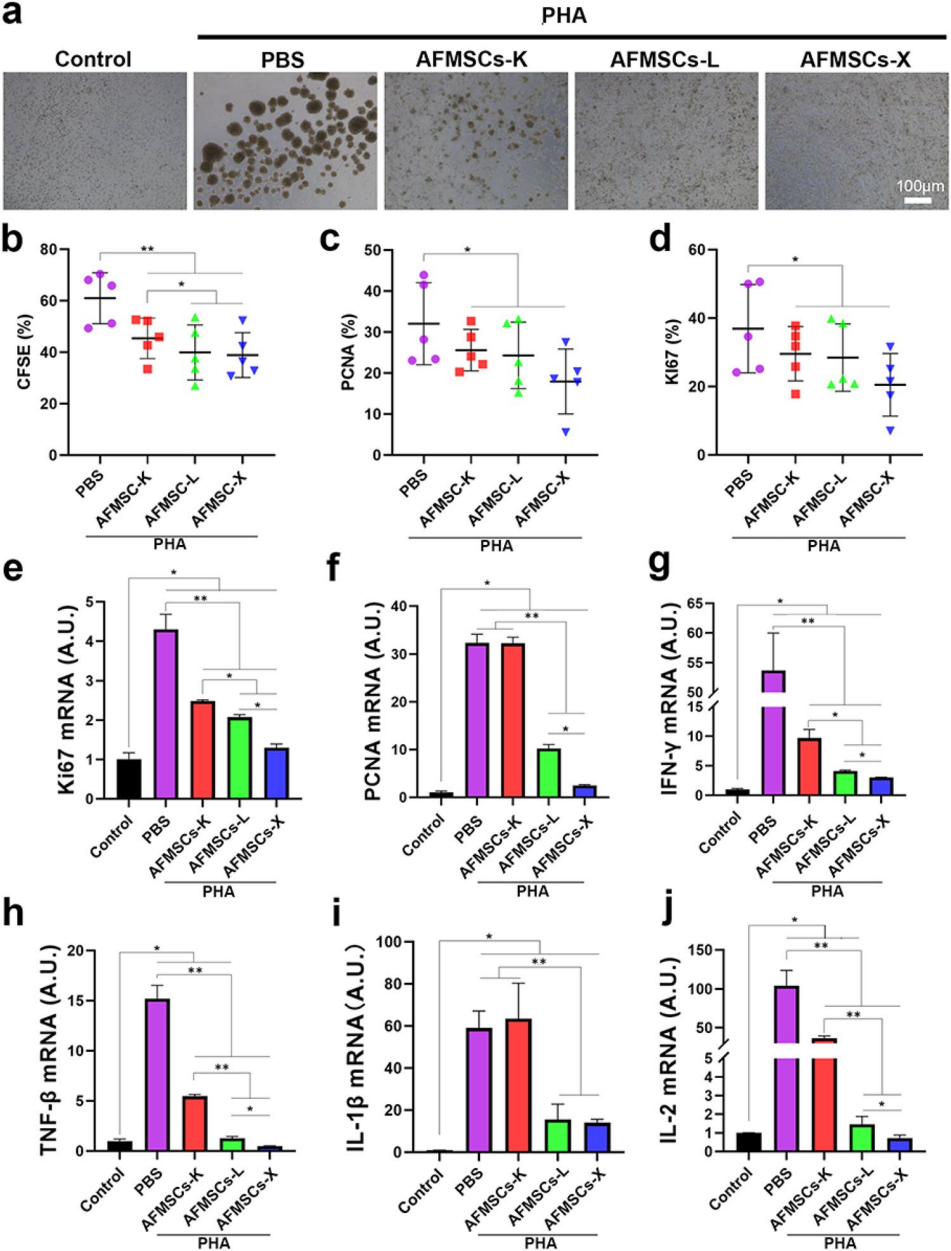
The AFMSCs-X shows a better immunosuppressive ability in vitro than the other two subtypes. **a** Bright-field to observe the inhibitory effects of different tissue-specific AFMSCs on the proliferation of PBMCs stimulated by PHA; **b**–**d** Percentage of positive CFSE, Ki67, and PCNA expression in different AFMSCs groups (*n* = 5, *p < 0.05, **p < 0.01); (e)-(f) mRNA expression levels of genes related to cellular proliferation (*Ki-67*, *PCNA*) were assessed (*n* = 3, *p < 0.05, **p < 0.01); (g)-(j) mRNA expression levels of genes encoding pro-inflammatory cytokines (*IFN-γ*, *TNF-β*, *IL-1β*, *IL-2*) (*n* = 3, *p < 0.05, **p < 0.01)

The immunomodulatory effects of AFMSCs were evaluated by human peripheral blood mononuclear cells (PBMCs) inhibition assay. AFMSCs inhibited PHA-induced aggregation and proliferation of PBMCs compared to PHA alone, with AFMSCs-X showing the strongest immunosuppressive ability followed by AFMSCs-L and AFMSCs-K (Fig. 5 a). This was confirmed by reduced CFSE-, Ki67- and PCNA-positive cells (Fig. 5 b-d and Supplementary Fig. 5) and downregulation of proliferation (*PCNA* and *Ki-67*) and inflammatory (*IFN-γ, TNF-β, IL-1β,* and *IL-2*) markers versus PHA (Fig. 5 e-j). Thus tissue-specific AFMSCs demonstrated immunosuppressive capabilities by suppressing PHA-induced activation.

### AFMSCs-X displayed remarkable protective effects on CLP-induced sepsis in mice

**Fig. 6:**
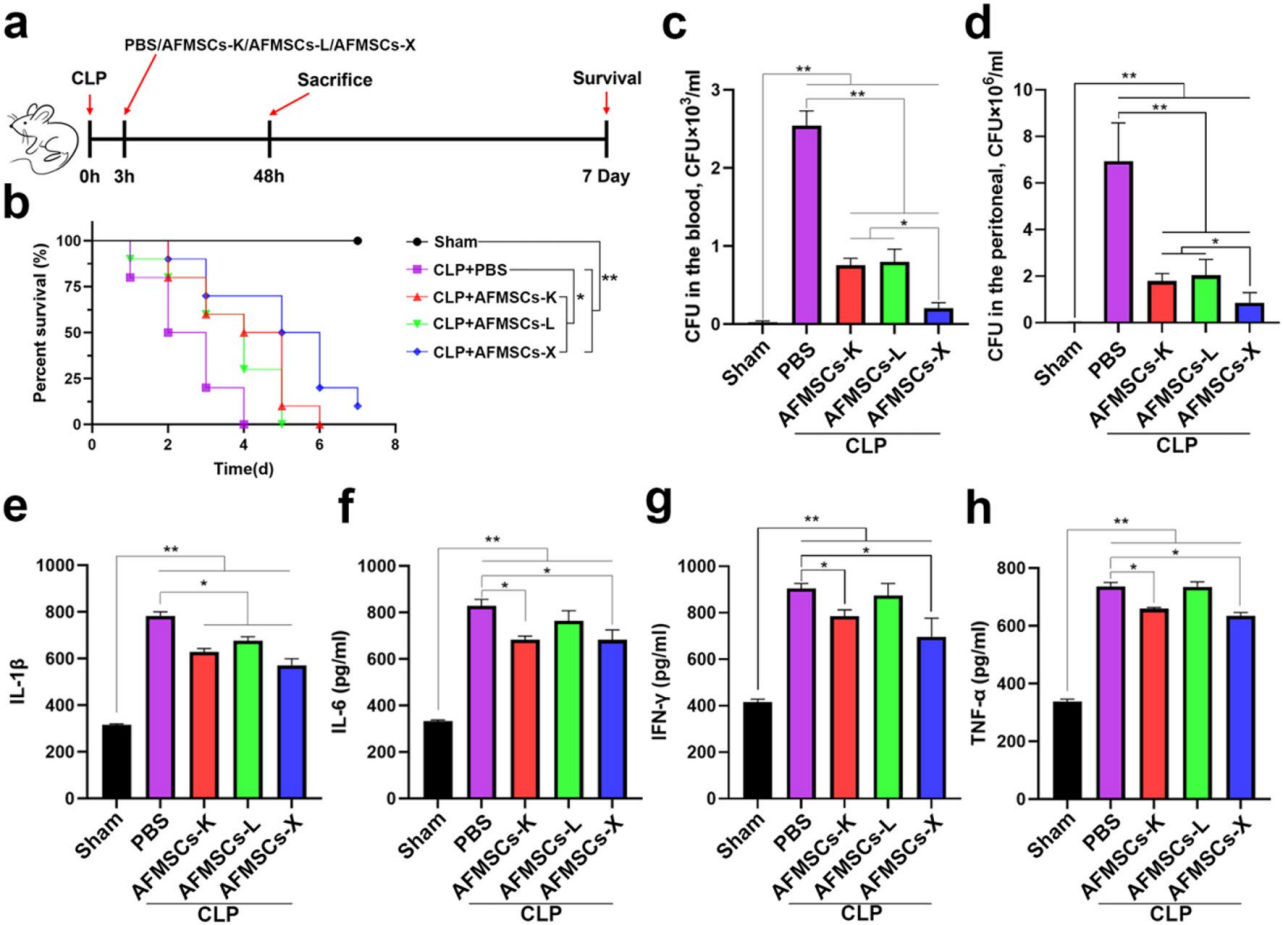
The AFMSCs-X shows better therapeutic efficacy than the other two subtypes in treating CLP-induced septic mice in vivo. **a** Sketch of making CLP-induced sepsis on mice and treated with AFMSCs; **b** Survival curve of mice in different experimental groups with AFMSCs treatment on day 7 post CLP induction (*n* = 10, *p < 0.05, **p < 0.01); **c**–**d** Quantification of blood and peripheral CFUs showed as the mean ± SD. (*n* = 3, *p < 0.05, **p < 0.01); (e)-(h) Serum plasma levels of pro-inflammatory cytokines (IL-1β, IL-6, IFN-γ and TNF-α) were assessed using ELISA immunoassay (*n* = 3, *p < 0.05, **p < 0.01)

A cecal ligation and puncture (CLP)-induced sepsis mouse model was used to investigate the protective effects of tissue-specific AFMSCs. Tissue-specific AFMSCs were injected via the tail vein 3 h after induction of CLP (Fig. 6 a). The survival rate of the CLP group rapidly decreased to 20% by 72 h after CLP induction. While mice treated with AFMSCs showed higher survival rates (Fig. 6 b). To further evaluate the efficacy of each AFMSC type in clearing bacteria, we quantified bacterial colony forming units (CFU) in blood and peritoneal fluid collected from AFMSC-treated mice 48 h after CLP induction. The AFMSCs-X treated group had the lowest bacterial CFU counts compared to AFMSCs-K, AFMSCs-L, and CLP groups (Fig. 6. c-d, Supplementary Fig. 6). Serum proinflammatory cytokines were measured to evaluate the systemic inflammatory response to AFMSCs administration. ELISA results showed CLP increased serum IL-1β, IL-6, IFN-γ, and TNF-α versus sham (Fig. 6e-h). AFMSCs-K and AFMSCs-X groups had significant decreases in these cytokines versus CLP, while the AFMSCs-L group only had decreased IL-1β (Fig. 6e). This demonstrates tissue-specific AFMSCs improved survival and reduced bacteremia in a CLP-induced sepsis mouse model, with AFMSCs-X demonstrating the most potent anti-inflammatory effects.

## Discussion

Although AFMSCs largely conform to MSC criteria in morphology, phenotype, and differentiation potential in vitro [13], they demonstrate superior proliferation and more robust differentiation potential than other adults MSCs [14, 15]. Additionally, they possess higher and more specific immunomodulatory abilities [4, 9, 10], and have several advantages over other types of MSCs in cell processing. Firstly, amniocentesis is safer and causes little pain for the donors compared to bone marrow aspiration and liposuction. Secondly, the culture of primary AFMSCs does not require the time-consuming process of tissue isolation and enzymatic dissociation, as they are suspended in the AF as single cells and rapidly expand into single-cell colonies. However, AFMSCs also show considerable heterogeneity, containing multiple cell subtypes derived from the developing fetus. Thus, it is urgent to find a method to reduce cellular heterogeneity of AFMSCs, determine the biological and molecular characterization of the main phenotypic subpopulation before any clinical use.

Amniotic fluid volume and composition change significantly during pregnancy, reflecting fetal physiology [8, 12]. AFMSCs’ proliferation and differentiation potential are influenced by donors’ gestational weeks [1]. Limited AFSCs exhibit ESCs-like characteristics during the first trimester, though obtaining samples during this stage is challenging [16]. Typically, amniocentesis is performed for prenatal genetic diagnosis from the 15th week. Our characterization of AF cell populations samples used from the 16th to 24th gestation weeks, corresponding to the second trimester. Term AF cells hold potential for cell therapy, requiring further research. In a 2020 study [4], we explored culture medium effects on AFMSCs isolation and cultivation during the second trimester. This study investigate AFMSCs heterogeneity and identifies tissue sources for various AF-derived stem cells (AFSCs) types, building on our previous research.

AFMSCs-K, the largest population, aligning with previous studies [9–11] (Fig. 1c), exhibits distinctive cell size, morphology and refractivity across groups (Fig. 1d). We posit that refractivity correlates with cell morphology and protein expression, supported by Roubelakis MG et al.’s finding of two distinct adherent cell types, SS-AF-MPCs and RS-AF-MPCs [7]. These cells were evaluated for MSC markers (Vimentin, N-cadherin and E-cadherin), with both types positive for Vimentin, while SS-AF-MPCs showed higher E- and N-cadherin expression. CD90 expression influenced morphology, proliferation and differentiation, potentially explaining the differences between the SS-AF-MPCs (CD90^high^) and RS-AFMPCs (CD90^low^) [7]. Similar studies by Pipino et al. revealed distinct proteomic profiles in Epithelial-like (E-like) and Fibroblast-like (F-like) AFMSCs, characterized by round/polyhedral and elongated/ spindle-shaped morphology, respectively, with HSB1 presence in F-like AFMSCs [5]. Our study aligns with these findings: (1) AFMSCs-X showed higher CD90, CD105 and CD117 levels, correlating with increased proliferation capacity. (2) AFMSCs-X exhibited morphology resembling SS-AF-MPCs/F-like phenotype from previous studies. AFMSCs-K and AFMSCs-L resemble the RS-AF-MPCs/E-like phenotype. However, further investigation is needed to uncover potential signaling pathways linking morphology and refraction.

The cell-surface antigenic profile of AFMSCs has been determined through flow cytometry and immunofluorescence staining. Previous studies have shown that AFMSCs obtained from early and second-trimester AF can express various ESCs markers such as OCT-4, SOX2, NANOG, SSEA4, and TRA1-81, which may raise the concern of teratoma formation post-transplantation [1]. However, in the present study, the immunofluorescence and flow cytometry results show that the AFMSCs mainly expressed SSEA4 with very weak expressed OCT4, SOX2, and NANOG. The potential reason for the discrepancy with the published reports is that OCT4, SOX2, and NANOG are highly expressed in freshly isolated AFMSCs and decreased in the expanding process. Although the three tissue-specific AFMSCs express a very low level of pluripotent markers, the AFMSCs are not tumorigenic and are confirmed with the spheroid formation assay in vitro and teratoma formation in vivo which ensure the safety of AFMSCs for translational application.

Additionally to AFMSCs, CD117(c-Kit)^+^/Lin^−^ amniotic fluid stem cells (AFSC) have been described [17, 18], they characterized a broadly multipotent which can be able to differentiation not only into mesoderm, but also in nonmesodermal lineages without tumorgenicity [1, 3, 6, 9, 18, 19]. CD117(c-Kit)^+^/Lin^−^ AFSC were an attractive candidate for regenerative medicine, but these cells are rarer (typically around 1% of live cells) and according to some, may be too much of a heterogeneous cell source[18], with high donor variations and therefore difficult to utilize for autologous cell therapy [2]. In our study, the subpopulation of AFMSCs-X expressed CD117 at a level of (19.31 ± 13.90) %, and AFMSCs-X was accounting for 9.05% of the total clones which is higher than CD117^+^ percentage. CD117^+^ AFSC exhibited variations in protein expression mainly occurring at early passages [5, 20], while AFMSCs-X have high proliferation, differentiation and immunomodulation potential in our study. It seems that AFMSCs-X have an even greater potential might be used clinically with specific properties.

Immunomodulatory capability endows the MSCs with great value for treating auto-immune diseases. AFMSCs have exerted a more substantial immunomodulatory effect on activated T cells than those of bone marrow MSCs, placenta MSCs, or UCMSCs [4, 21]. In addition, transplanted AFMSCs can respond to inflammatory cytokines (e.g., IFN-γ and TNF-α) and exert an immunosuppressive effect for regulating the proliferation and activation of immune cells [3]. In this study, all three tissue-specific AFMSCs showed excellent immunomodulatory ability in vitro. In particular, AFMSCs-X had the best immunosuppressive power than other two subtypes.

So far, few studies have examined the abilities of AFMSCs in the context of anti-sepsis therapy [22–24]. The present study is the first to comprehensively examine the effects of tissue-specific AFMSCs in treating mice with CLP-induced sepsis. AFMSCs-K and AFMSCs-X significantly reduced the levels of pro-inflammatory cytokines IL-1β, IL-6, IFN-γ, and TNF-α. Surprisingly, they also showed direct anti-microbial effects. Of note, tissue-specific AFMSCs significantly reduced the number of bacterial CFUs in blood and peritoneal fluid collected 24 h after CLP. The mechanism of bacteria scavenging by AFMSCs may involve activation of phagocytic monocytes in the blood [25, 26] and secretion of two anti-microbial peptide (LL-37) [27] and ferritin [28]. Back to the main point, AFMSCs exerted anti-inflammatory and bacteria-scavenging effects in the CLP-induced mice, indicating their potential value for treating inflammatory diseases. Never least, recognition and administration of the tissue-specific AFMSCs may represent a reliable approach to improving the consistency and efficacy of AFMSCs for translational research and application.

## Conclusions

We introduce a novel method for categorizing AFMSCs as three subtypes based on single cell cloning and tissue-specific gene expression pattern, much more advanced than the contemporary methods based on morphologic characteristics and surface markers. Furthermore, we evaluated the phenotype and anti-inflammatory effects of all the AFMSC subtypes in vitro and in CLP-induced sepsis mice. The results showed that this tissue-specific AFMSCs possessed individual biological characteristics and showed anti-inflammatory and antibacterial effects in CLP mice. Our approach may increase the homogeneity of AFMSCs and improves the consistency and stability of AFMSCs, accelerating the application of AFMSCs for translational research.

## Methods

### Cell source

The AF used in this study was donated by ten female patients who underwent amniocentesis without fetal sonographic structural abnormalities in their second trimester of pregnancy (16–24 week) at The Third Affiliated Hospital of Guangzhou Medical University (Guangzhou, China). Meanwhile, part of the patients’ information and their karyotype results are shown in Table 1. All donors provided their written informed consent. This study was reviewed and approved by the Ethics Committee and the Institutional Review Board of the Third Affiliated Hospital of Guangzhou Medical University (No. 2021–023).

### Cell culture

We performed a series of experimental single cell clonal culture of AFMSCs. Routinely we cultured a 10 mL AF cell pellet in a 25 cm^2^ cell culture dish and approximately 5–40 cell clones formed and used for clinical karyotype detection. In this study, we reduced the volume of AF to 5 mL and centrifuged at 300×g for 5 min at room temperature (RT). Then the cell pellet was suspended in 9 mL commercial AFMSCs medium (BI), transferred into 10-cm Petri dishes, which was enough for AFMSCs to form single cell clones. After 7–9 days, clones which are uniform and have very clear boundaries were defined as a single cell clones. These clones were treated with 0.05% trypsin–EDTA (Gibco), mechanically separated and transferred into 6-well plates for subsequent expansion. All the assays were conducted with AFMSCs of passage 3 unless there is a specific notation.

### Population doubling time (PDT) assay

Tissue-specific AFMSCs (P2–P6) were cultured to determine the population doubling time (PDT). Cells were stained with trypan blue (Thermo) using a cell counter (Thermo). Approximately, 2 × 10^5^ cells were seeded in a 10-cm^2^ culture dish and PDT was calculated using an online tool based on the final cell number (http://www.doubling-time.com/Compute.php).

### SA-β-galactosidase staining

Cellular senescence was assessed using a β-Galactosidase Staining Kit (Beyotime). Briefly, culture media was removed and cells were washed with PBS, cells were fixed with 4% paraformaldehyde (PFA, v/v) at RT for 15 min. Fixation solution was removed and cells were rinsed 3 times with PBS for 5 min each. Cells were then treated with 1 mL staining solution and incubated overnight at 37 °C. β-Galactosidase staining was then observed using a bright field inverted microscope (Leica).

### Flow cytometry

About 3 × 10^6^ cells AFMSCs were incubated for 30 min at RT with fluorescent-conjugated antibodies against CD19-FITC (Biolegend, Cat# 306,204), HLA-DR-APC (Biolegend, Cat# 307,610), CD34-FITC (BD, Cat# 560,942), CD45-APC (BD, Cat# 555,485), CD11b-APC (Biolegend, Cat# 301,310), CD73-APC (Biolegend, Cat# 344,005), CD44-PE/CY7 (Biolegend, Cat# 103,029), CD29-PE (Biolegend, Cat# 303,003), CD117-APC (Biolegend, Cat# 313,206), CD105-PE (Biolegend, Cat# 800,503), CD90-PerCP/Cyanine5.5 (Biolegend, Cat# 328,117), PCNA-PE (Biolegend, Cat# 307,908) and Ki-67-APC (Biolegend, Cat# 350,513). AFMSCs were then rinsed twice with PBS and analyzed by flow cytometry (Thermo).

### Cell differentiation assays

AFMSCs reached 70–80% confluence, then differentiated for 21 days in inducing media. The osteogenic differentiation medium consisted of DMEM, 50 μM L-corbic acid (MedChem Express), 100 nM dexamethasone (Sigma), and 10 mM β-glycerophosphate disodium salt hydrate (Sigma). The chondrogenic differentiation medium consisted of DMEM, 1 μM dexamethasone (Sigma), 50 μM L-ascorbic acid (Med Chem Express), 500 μM sodium pyruvate solution (Sigma), 10 μg/L TGF-β1 (Novoprotein) and 1% insulin–transferrin–sodium selenite media supplement (ITS) (Thermo). The adipogenic differentiation medium was DMEM supplemented with 0.5 mM IBMX (Sigma), 1 μM dexamethasone (Sigma), 10 μM insulin (Meilunbio) and 200 μM indometacin (Sigma). On day 21, trilineage differentiation were stained with Alizarin red S, Toluidine blue and Oil Red O, respectively.

### Immunosuppression of PHA-stimulated PBMCs

AFMSCs were pre-treated with 10 µg/mL mitomycin C (Med Chem Express) for 3 h. PBMCs isolated from healthy donors via Ficoll-Paque (GE) were labeled with the fluorescent dye 5, 6-carboxyfluorescein diacetate succinimidyl ester (CFDA-SE) (Invitrogen). AFMSCs and PBMCs were then co-cultured at a 1:5 ratio for 72 h in RPMI-1640 (CORNING) supplemented with 30% FBS (Gibco) and 100 μg/mL PHA (Dahui Biotechnology). PBMCs proliferation were evaluated by measuring CFSE fluorescence via flow cytometry, and mRNA expression was examined using RT-qPCR.

### Care and housing of experimental animals

All animal procedures were approved by the Ethics Research Committee of The Third Affiliated Hospital of Guangzhou Medical University. C57BL/6 mice (weighing 18–20 g, 5–6 weeks) were purchased from the Guangdong Medical Laboratory Animal Centre (Guangzhou, China), housed under standard conditions with a 12 h light–dark cycle, and are free access to food and water.

### Sepsis induction and treatment

The mice were randomly assigned to five experimental groups (nine mice per group): sham group, CLP group, and 3 treatment groups (CLP + AFMSCs-K, CLP + AFMSCs-L, or CLP + AFMSCs-X). Three hours following CLP, mice in the treatment group received an intravenous injection of approximately 3 × 10^5^ AFMSCs suspended in 150 μL PBS via the caudal vein. The sham and CLP groups received PBS only. The 7-day survival rates were determined, peripheral blood and peritoneal fluid bacterial CFU counts and peripheral blood serum were collected at 48 h. At the end of the study, mice were euthanized by decapitation.

### Enzyme-linked immunosorbent assay (ELISA)

Cytokine levels in mouse sera were analyzed using ELISA immunoassays (MEIMIAN), including IL-1β, IL-6, IFN-γ and TNF-α. In addition, optical density (OD) was measured at 450-nm wavelength using an ELISA plate reader (BioTek).

### Bacterial colony forming unit (CFU) counts

Peripheral blood (50 μL) and peritoneal fluid (5 μL) were diluted 20- or 20,000-fold with PBS, respectively. The diluted samples were plated on blood agar plates (HuanKai Microbial) and incubated in a carbon dioxide incubator at 37 °C for 24 h. Then, bacterial CFU was imaged using a microscope and quantified using Image J.

### Quantitative real-time polymerase chain reaction (RT-qPCR)

Total RNA was extracted using TRIzol™ (Invitrogen). cDNA was synthesized using PrimeScript™RT Kit (Takara). qPCR was performed on a StepOne™ Real-Time PCR System using SYBR® Premix Ex Taq™ II (Takara) and analyzed using ViiA7™ System software (Thermo). The primers used in this procedure are listed in Supplementary Table 1. The mRNA expression was normalized to β-Actin mRNA, and relative mRNA levels were calculated using the ∆∆CT method.

### Statistical analysis

All experiments were performed in triplicate with quantitative results expressed as mean ± SD. Statistical comparisons between two groups were conducted using unpaired, two-tailed Student’s t-tests. Log-rank (Mantel-Cox) tests were used to analyze survival data. *P* value < 0.05 was defined as statistical significance. GraphPad Prism 8 were used for statistical analyses.

### Supplementary Information

Below is the link to the electronic supplementary material.

Supplementary file1 (TIF 5643 kb)

Supplementary file2 (TIF 3981 kb)

Supplementary file3 (TIF 7295 kb)

Supplementary file4 (TIF 4755 kb)

Supplementary file5 (TIF 6696 kb)

Supplementary file6 (TIF 8755 kb)

Supplementary file7 (DOCX 13 kb)

Supplementary file8 (DOCX 12 kb)
