# Supplementary figures and images for: Tissue-specific populations from amniotic fluid-derived mesenchymal stem cells manifest variant in vitro and in vivo properties
Source: Hum Cell. 2023 Dec 12;37(2):408–19. doi: 10.1007/s13577-023-01008-z (PMC10891244; doi:10.1007/s13577-023-01008-z)

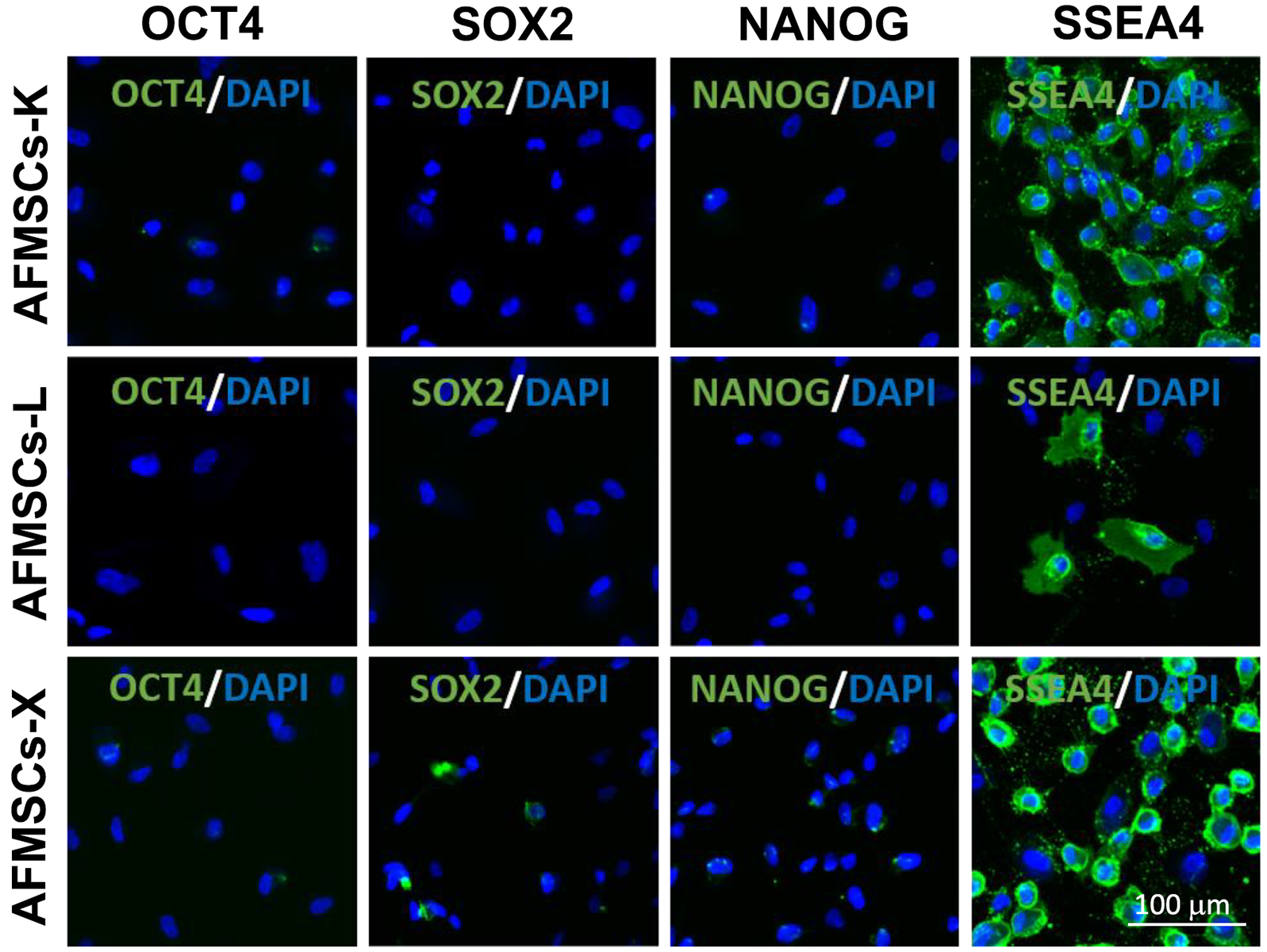

Supplement: Supplementary file 1 — Supplementary file1 (TIF 5643 kb) [file 13577_2023_1008_MOESM1_ESM.tif]

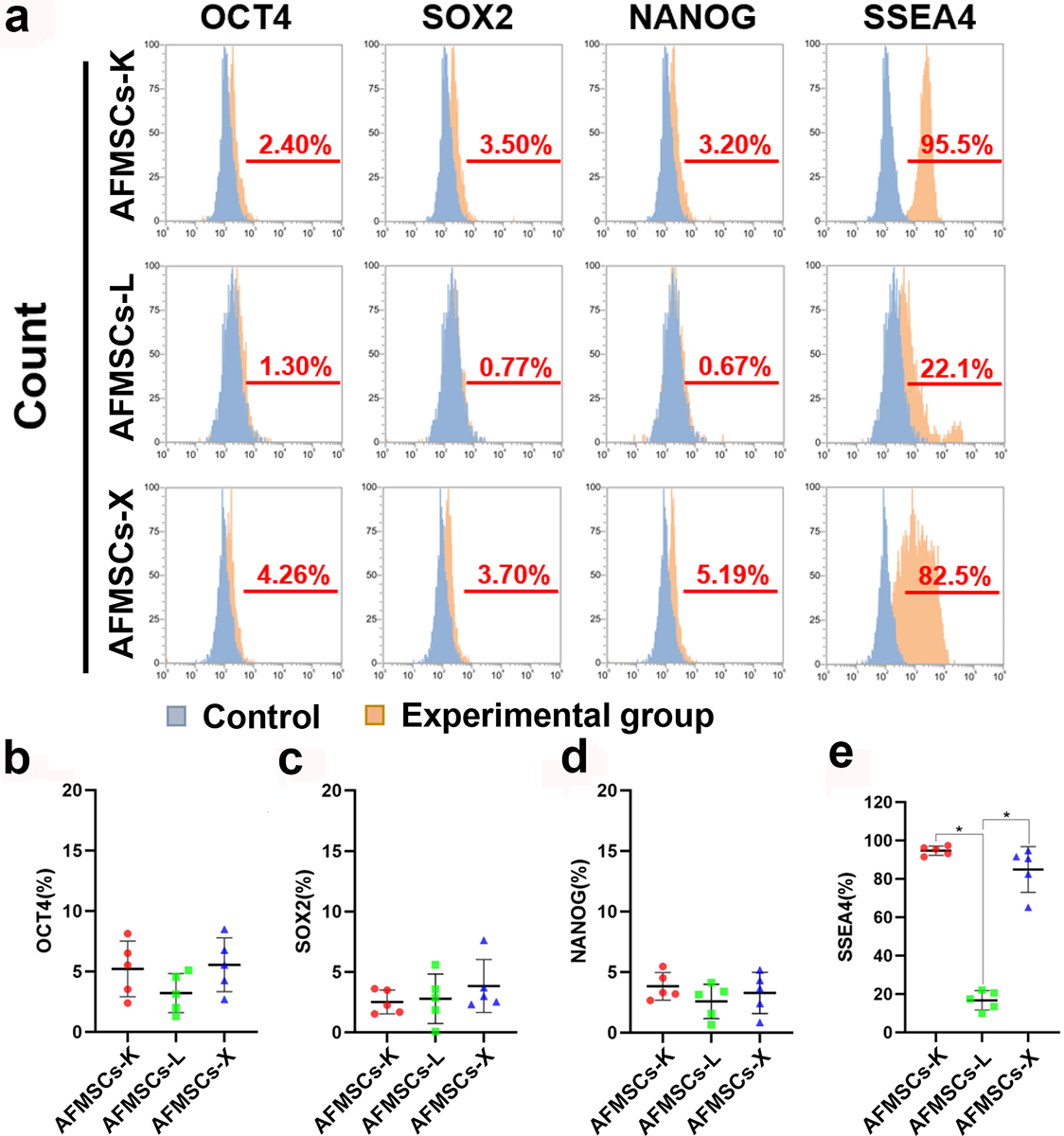

Supplement: Supplementary file 2 — Supplementary file2 (TIF 3981 kb) [file 13577_2023_1008_MOESM2_ESM.tif]

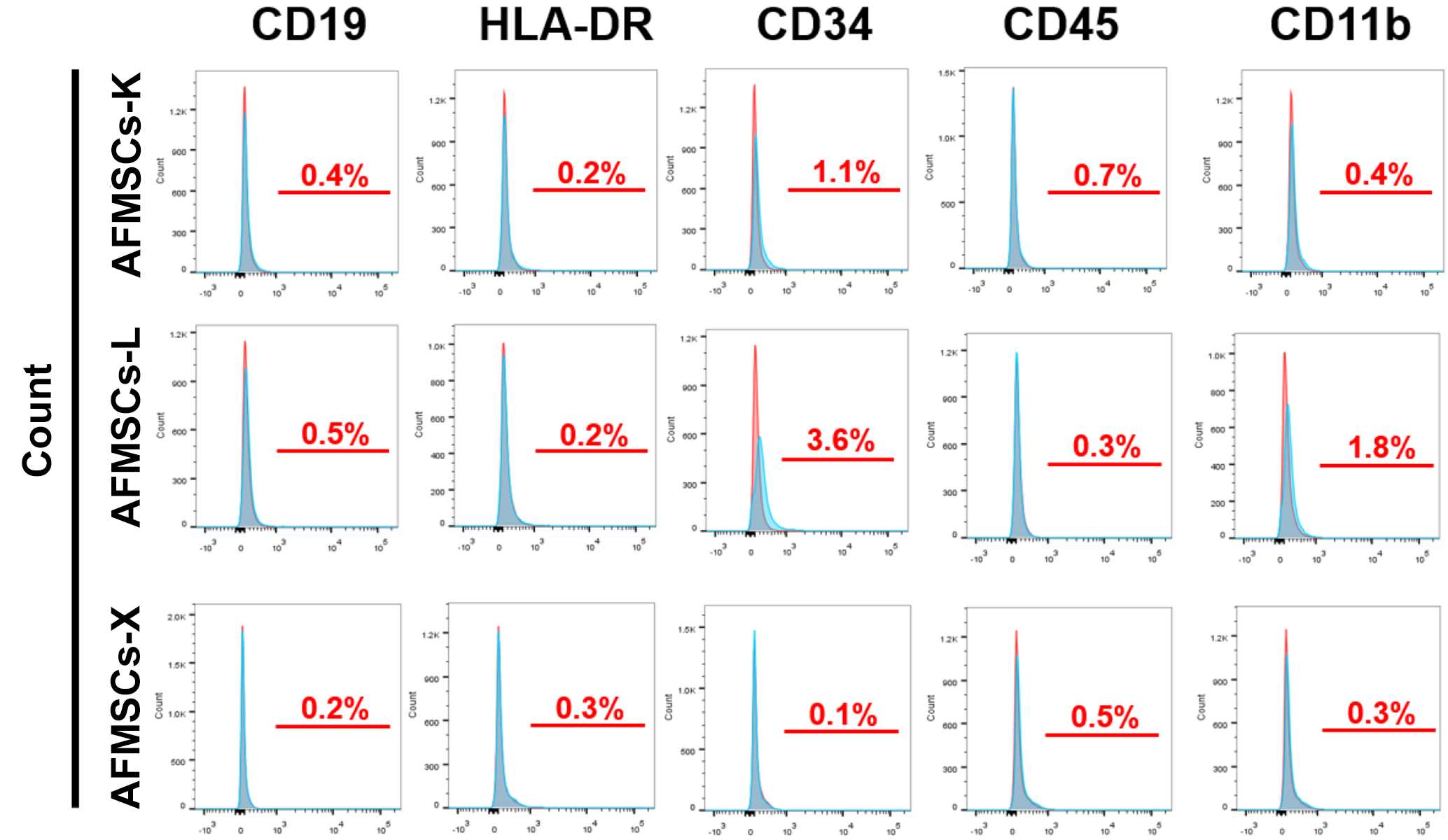

Supplement: Supplementary file 3 — Supplementary file3 (TIF 7295 kb) [file 13577_2023_1008_MOESM3_ESM.tif]

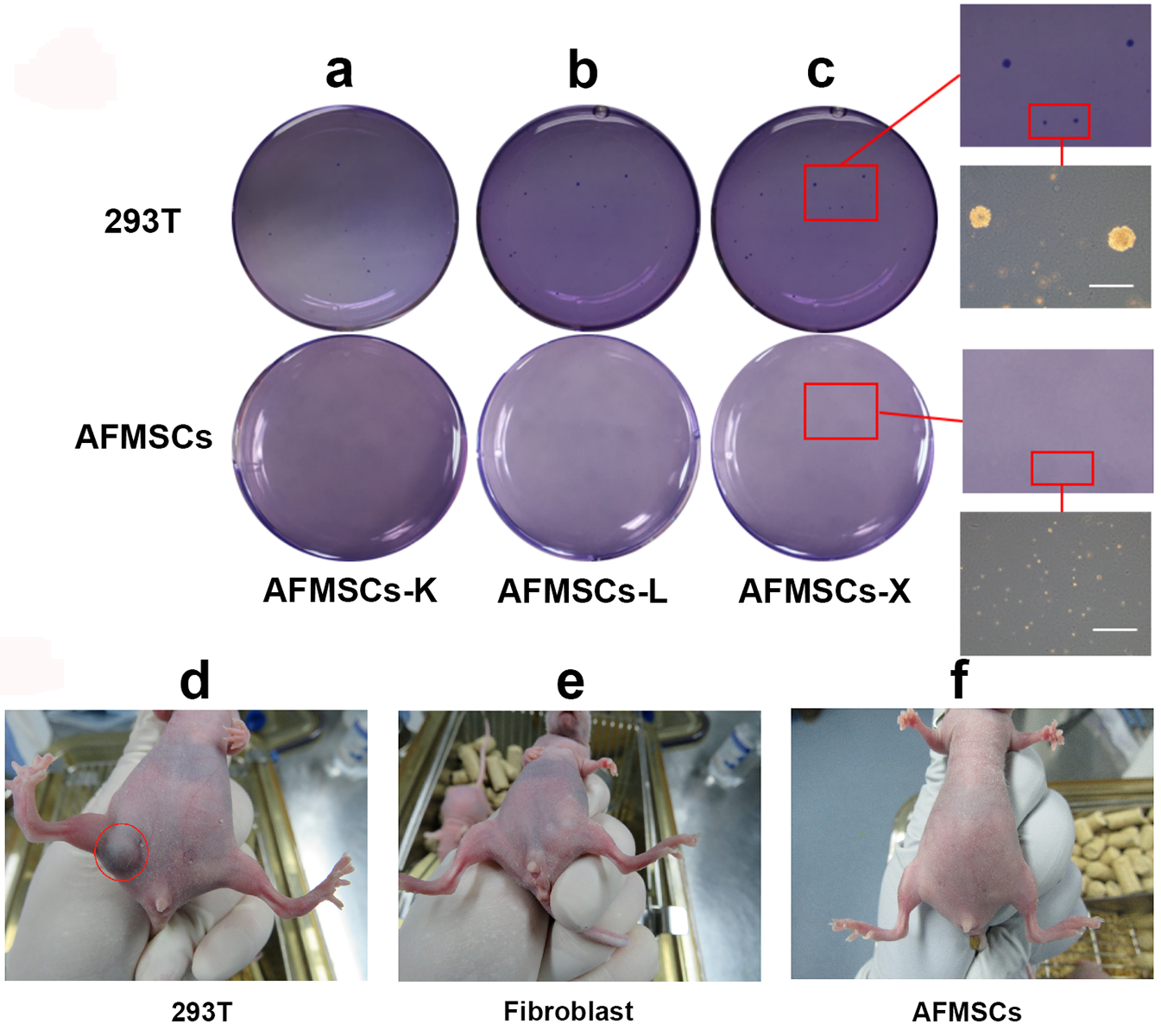

Supplement: Supplementary file 4 — Supplementary file4 (TIF 4755 kb) [file 13577_2023_1008_MOESM4_ESM.tif]

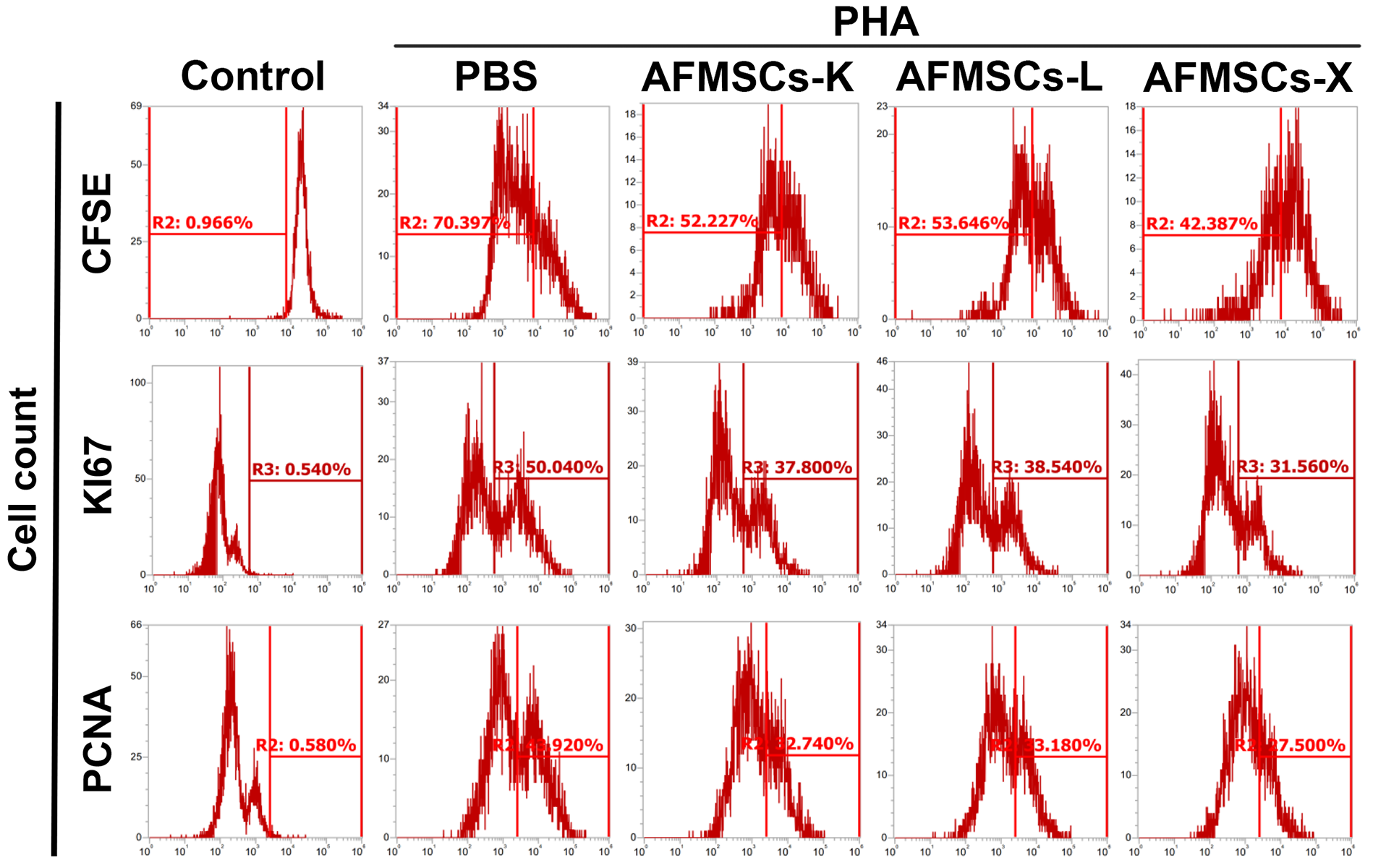

Supplement: Supplementary file 5 — Supplementary file5 (TIF 6696 kb) [file 13577_2023_1008_MOESM5_ESM.tif]

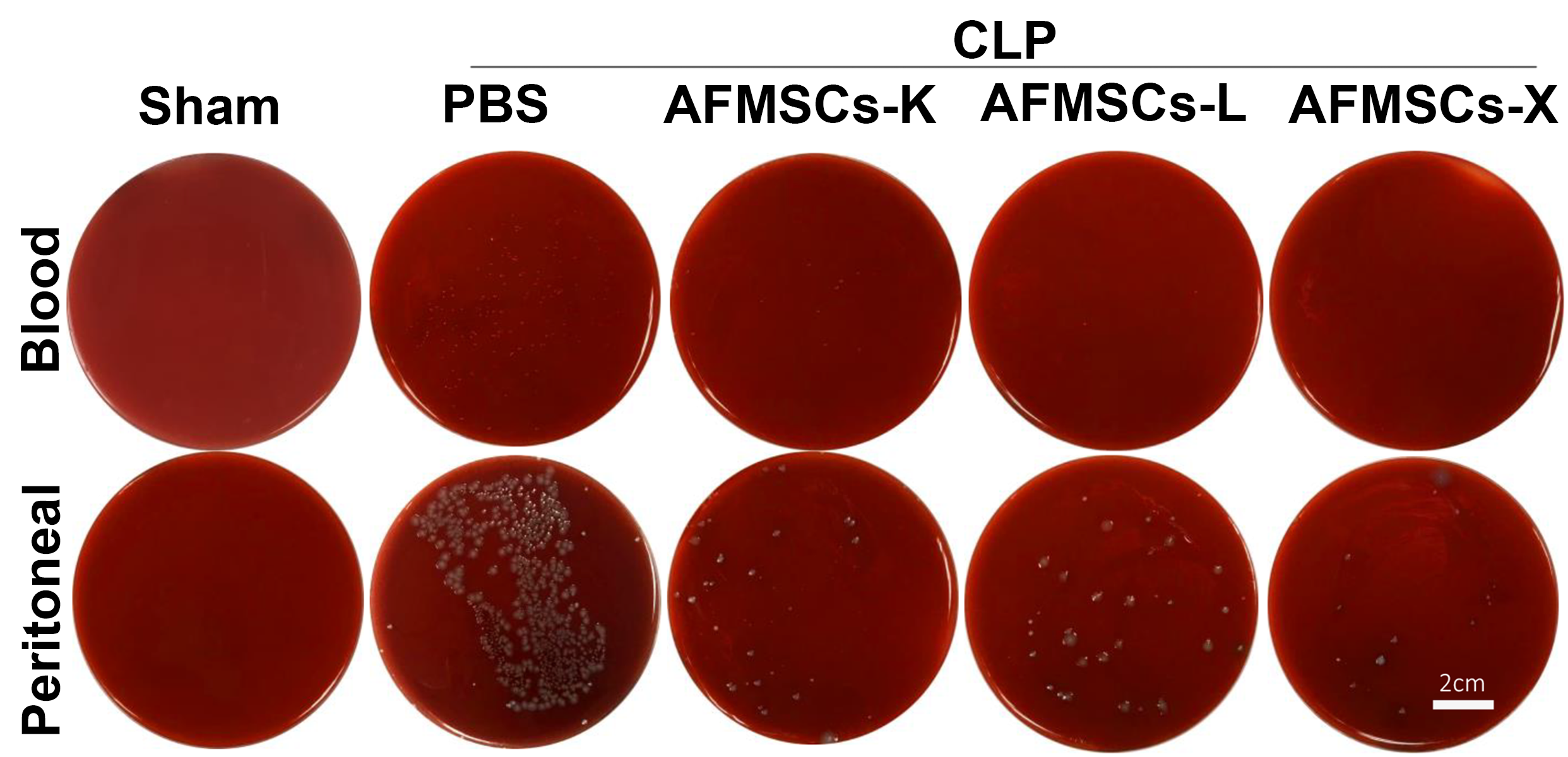

Supplement: Supplementary file 6 — Supplementary file6 (TIF 8755 kb) [file 13577_2023_1008_MOESM6_ESM.tif]
