# Supplementary material for: Tissue-specific populations from amniotic fluid-derived mesenchymal stem cells manifest variant in vitro and in vivo properties
Source: Hum Cell. 2023 Dec 12;37(2):408–19. doi: 10.1007/s13577-023-01008-z (PMC10891244; doi:10.1007/s13577-023-01008-z)
Supplement: Supplementary file 7 — Supplementary file7 (DOCX 13 kb) [file 13577_2023_1008_MOESM7_ESM.docx]

**Supplementary materials and methods**

**S1. Immunofluorescence labeling**

P3 AFMSCs were fixed using 4% PFA for 20 minutes and then permeabilized using 0.5% TritonX-100 for 10 min at RT. Next, AFMSCs were incubated in 5% bovine serum albumin (BSA) for 1 h, and then incubated overnight at 4°C with the following primary antibodies: Mouse anti-OCT4 (Abcam, Cat# ab181557), Rabbit anti-SOX2 (Abcam, Cat# ab79351), Mouse anti-SSEA4 (Abcam, Cat# ab16287), and Rabbit anti-Nanog (Abcam, Cat# ab214549). Afterwards, AFMSCs were incubated for 1 h at RT with the following secondary antibodies: Goat anti-rabbit IgG H&L (Invitrogen, Cat# A11008, Alexa Fluor® 488) or Donkey anti-mouse IgG H&L (Invitrogen, Cat# A21202, Alexa Fluor® 488). Nuclei were stained using 1 μg/mL DAPI (Roche, Cat# 28718-90-3) for 10 min and then observed using confocal microscope (Nikon, C2Si, Nis Elements-C software).

**S2. Flow cytometry**

P3 AFMSCs were digested using trypsin/EDTA and counted. About 3×10^6^ cells AFMSCs were incubated for 30 min at room temperature (RT), using the following fluorescent antibodies: CD19-FITC (Biolegend, Cat# 306204), HLA-DR-APC (Biolegend, Cat# 307610), CD34-FITC (BD Biosciences, Cat# 560942), CD45-APC (BD Biosciences, Cat# 555485), CD11b-APC (Biolegend, Cat# 301310), Mouse anti-OCT4 (Abcam, Cat# ab181557), Rabbit anti-SOX2 (Abcam, Cat# ab79351), Mouse anti-SSEA4 (Abcam, Cat# ab16287), Rabbit anti-Nanog (Abcam, Cat# ab214549), and Mouse anti-Tra1-81 (Abcam, Cat# ab16289). AFSCs were then washed twice with PBS and analyzed using a flow cytometer (Attune™ NxT Acoustic Focusing Cytometer, A24863, Thermo Fisher Scientific). The same voltage parameters for forward scatter (FSC) and side scatter (SSC) were used in all the assays.

**S3. Soft Agar Colony Formation Assay of different tissue-specific AFMSCs**

Firstly, 1% noble agar solution and culture medium was mixed with equal volume, added 1.5 mL agar mixture to cover the 6-well plate, and placed the plate in the culture hood to solidify at RT, for 30 min. Secondly, plating the upper layer of agar containing about 5000 cells (each well will receive 0.75 mL cell suspension, 0.75 mL 0.6% agar solution). The time required for adequate colony formation varies for each cell line, in the current experiment, 293T clones appeared on day 21, and tissue-specific AFMSCs clone was not to be detected until 30 days. A layer of growth medium (100 μL) was added twice weekly over the upper layer of agar to prevent desiccation. Tumor colones were stained using 1mg/mL nitroblue tetrazolium chloride solution (Roche, Cat# 11383213001) which was added to the 6-well plate well and incubated plates overnight at 37 °C. Once colonies are stained, take photographs of wells using inverted microscope (Leica, DMi 1).

**S4. Teratoma formation *in vivo***

P3 tissue-specific AFMSCs were cultured for 3-4 days were treated with 0.05% trypsin-EDTA (Gibco, Cat# 25300054), collected in EP tube and centrifuged, resuspended in AFCs culture medium containing 50% Matrigel (Corning, Cat# 354277). About 5×10^6^ AFSMCs were injected into NOD-SCID mice subcutaneously. After 8 weeks, teratomas were checked in 293T, fibroblasts and AFMSCs groups.
