# Supplementary material for: Tissue-specific populations from amniotic fluid-derived mesenchymal stem cells manifest variant in vitro and in vivo properties
Source: Hum Cell. 2023 Dec 12;37(2):408–19. doi: 10.1007/s13577-023-01008-z (PMC10891244; doi:10.1007/s13577-023-01008-z)
Supplement: Supplementary file 8 — Supplementary file8 (DOCX 12 kb) [file 13577_2023_1008_MOESM8_ESM.docx]

Supplementary Table 1. Primers sequences used in RT-qPCR.

| Genes | Forward (5′‐3′) | Reverse (5′‐3′) |
| --- | --- | --- |
| *KSP* | AGCCTATCCACCTGGCAGAGAA | TCTGGTCACGTAGAGGTTTCCC |
| *NKX2.1* | CAGGACACCATGAGGAACAGCG | GCCATGTTCTTGCTCACGTCCC |
| *P16* | CTCGTGCTGATGCTACTGAGGA | GGTCGGCGCAGTTGGGCTCC |
| *P21* | AGGTGGACCTGGAGACTCTCAG | TCCTCTTGGAGAAGATCAGCCG |
| *P53* | CCTCAGCATCTTATCCGAGTGG | TGGATGGTGGTACAGTCAGAGC |
| *RUNX2* | CCCAGTATGAGAGTAGGTGTCC | GGGTAAGACTGGTCATAGGACC |
| *ALP* | GCTGTAAGGACATCGCCTACCA | CCTGGCTTTCTCGTCACTCTCA |
| *SOX9* | AGGAAGCTCGCGGACCAGTAC | GGTGGTCCTTCTTGTGCTGCAC |
| *COMP* | GGAGATGCTTGTGACAGCGATC | TGAGTCCTCCTGGGCACTGTTA |
| *ADIPO* | CAGGCCGTGATGGCAGAGATG | GGTTTCACCGATGTCTCCCTTAG |
| *LPL* | CTGCTGGCATTGCAGGAAGTCT | CATCAGGAGAAAGACGACTCGG |
| N-Cadherin | CCTCCAGAGTTTACTGCCATGAC | GTAGGATCTCCGCCACTGATTC |
| *SNAI2* | ATCTGCGGCAAGGCGTTTTCCA | GAGCCCTCAGATTTGACCTGTC |
| *SNAI3* | TGCACCTGCAAGATCTGTGGCA | AAGGTTGGAGCGGTCGGCAAAG |
| *Ki-67* | CGACCCTACAGAGTGCTCAACAA | CTTGTCAACTGCGGTTGCTCCTT |
| *PCNA* | CCACTCTCTTCAACGGTGACACT | CATCCTCGATCTTGGGAGCCAA |
| *INF-γ* | CCAAGTGATGGCTGAACTGTCG | GCAGGCAGGACAACCATTACTG |
| *TNF-β* | CACCTCCCTGAACCATCCCTGAT | CTCCATGTGCCTGCTCTTCCTCT |
| *IL-1β* | CCACAGACCTTCCAGGAGAATG | GTGCAGTTCAGTGATCGTACAGG |
| *IL-2* | AGAACTCAAACCTCTGGAGGAAG | GCTGTCTCATCAGCATATTCACAC |
